# The Role of Serotonin in Breast Cancer Stem Cells

**DOI:** 10.3390/molecules26113171

**Published:** 2021-05-26

**Authors:** William D. Gwynne, Mirza S. Shakeel, Adele Girgis-Gabardo, John A. Hassell

**Affiliations:** 1 Department of Surgery, McMaster University, Hamilton, ON L8S 4L8, Canada; gwynnewd@mcmaster.ca; 2 Department of Biochemistry and Biomedical Sciences, McMaster University, Hamilton, ON L8S 4L8, Canada; shahbaz.shakeel3@gmail.com (M.S.S.); gabardoa@mcmaster.ca (A.G.-G.)

**Keywords:** breast cancer, breast cancer stem cells, serotonin, serotonergic system antagonists, G-protein receptors, kinase signaling cascades

## Abstract

Breast tumors were the first tumors of epithelial origin shown to follow the cancer stem cell model. The model proposes that cancer stem cells are uniquely endowed with tumorigenic capacity and that their aberrant differentiation yields non-tumorigenic progeny, which constitute the bulk of the tumor cell population. Breast cancer stem cells resist therapies and seed metastases; thus, they account for breast cancer recurrence. Hence, targeting these cells is essential to achieve durable breast cancer remissions. We identified compounds including selective antagonists of multiple serotonergic system pathway components required for serotonin biosynthesis, transport, activity via multiple 5-HT receptors (5-HTRs), and catabolism that reduce the viability of breast cancer stem cells of both mouse and human origin using multiple orthologous assays. The molecular targets of the selective antagonists are expressed in breast tumors and breast cancer cell lines, which also produce serotonin, implying that it plays a required functional role in these cells. The selective antagonists act synergistically with chemotherapy to shrink mouse mammary tumors and human breast tumor xenografts primarily by inducing programmed tumor cell death. We hypothesize those serotonergic proteins of diverse activity function by common signaling pathways to maintain cancer stem cell viability. Here, we summarize our recent findings and the relevant literature regarding the role of serotonin in breast cancer.

## 1. Introduction

Breast cancer is the second most common cancer in women and the leading cause of cancer-related deaths worldwide [1]. Despite increased screening and new targeted therapies, the global incidence of breast cancer continues to increase [2]. Moreover, cytotoxic therapies used to treat breast cancer frequently do not achieve long lasting remissions. In fact, roughly 30% of women diagnosed with invasive breast cancer relapse and 90% of these individuals die from metastases. Hence, new therapies are needed that ensure long lasting remissions.

### 1.1. Breast Cancer Clinical and Molecular Subtypes

Breast tumors were first classified based on their hormone receptor status (estrogen (ER) and/or progesterone receptors (PR)), whether they overexpressed HER2, or did not express either ER, PR, or HER2, termed triple-negative breast cancer (TNBC) [3]. Global gene expression profiling was subsequently used to molecularly classify breast tumors into various subtypes: luminal A (ER+ and/or PR+/−); luminal B (ER+ and/or PR+/−) and either HER2 overexpressing or not; basal-like (triple negative breast cancers (TNBC), do not express hormone receptors nor overexpress HER2; and normal-like. Luminal A tumors comprise a low fraction of proliferating cells (defined by Ki67-positive cells), whereas luminal B tumors are composed of a high fraction of dividing tumor cells. HER-2 overexpressing breasts tumors do not express ER or PR or express them at low levels. Normal-like tumors are similar to luminal A tumors. The prognosis for patients with breast cancer is linked to their subtype in the order from best to worst prognosis: luminal A; normal-like; luminal B, HER2-overexpressing; and TNBC. Therapies are tailored for patients consistent with their breast tumor subtype. For example, patients with HER-2 overexpressing or ER+ breast tumors are treated with monoclonal antibodies or small molecules that target these receptors, or in the case of ER+ tumors, block the synthesis of estrogen. Unfortunately, resistance to these drugs often develops in patients.

### 1.2. Breast Cancer Stem Cells

**Figure 1:**
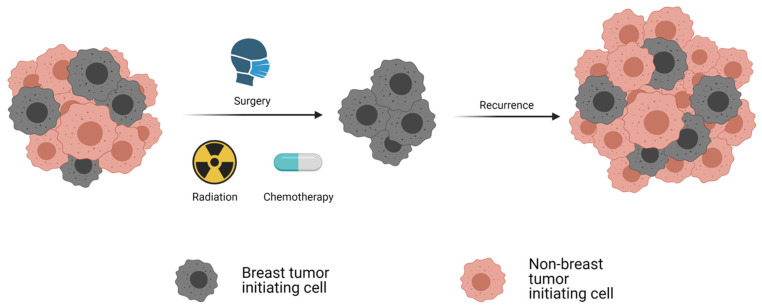
The breast cancer stem cell model for disease recurrence. Schematic conveying the clinical implications of therapy-resistant BTIC. Standard of care consisting of surgery and cytotoxic therapies principally eradicate non-BTIC. BTIC remain dormant until seeding local or distant disease recurrences.

Breast cancer recurrence has been attributed to breast cancer stem cells [4], which are functionally defined by their capacity to initiate tumor growth following their transplantation into mice and are consequently also termed breast tumor-initiating cells (BTICs) [5]. BTIC are refractory to cancer therapies, seed metastases, and account for breast cancer recurrence (Figure 1). Analyses of the mammary tumors from various transgenic mouse models of breast cancer revealed that like human breast tumors, these also follow the cancer stem cell model [6,7,8,9]. The model proposes that genetic or epigenetic changes in tissue-specific cells yield tumor cells with stem cell-like properties, including the capacity for limitless self-renewal and differentiation [10]. The cellular heterogeneity of tumors is due to the aberrant differentiation of the stem-like tumor cells. Hence, breast tumors comprise a cellular hierarchy of BTIC at their apex and non-tumorigenic proliferating and differentiating progeny of these cells at their base. Whether BTIC originate from normal breast stem cells or whether their frequency differs among breast tumors of distinct subtypes has not yet been unequivocally established.

### 1.3. Epithelial to Mesenchymal Transition and BTIC, Therapeutic Implications

Studies during the last decade demonstrate that the induction of an epithelial to mesenchymal transition (EMT) can endow “non-tumorigenic” breast tumor cells with BTIC activity, implying that breast tumor cells transition between non-tumorigenic and tumorigenic states [11,12,13]. These findings have therapeutic implications [14]. Cytotoxic therapies eradicate the proliferating non-tumorigenic progeny of BTIC, which comprise the majority of cells populating tumors. Indeed, the fraction of BTIC increases in tumors after neoadjuvant chemotherapy due to their resistance to cytotoxic drugs and the loss of the non-tumorigenic tumor cells (Figure 1) [15,16]. Hence, tumors regress after standard therapies, but they often recur. Long-lasting breast cancer remissions require new therapies that eradicate both BTIC and their non-tumorigenic progeny, which are a potential reservoir of these cells.

## 2. Discovery of BTIC Targeting Small Molecules

### 2.1. Identification of an Abundant Source of BTIC

To identify BTIC targeting agents requires an abundant source of these cells. To this end, the frequency of BTIC in mouse mammary tumors of three transgenic models of breast cancer was established by our group using limiting dilution tumor cell transplantation experiments. Surprisingly, the mammary tumors from all the models (MMTV-Neu (HER2 overexpressing), MMTV-polyomavirus middle tumor antigen, and MMTV-beta (β)-catenin) comprised a high BTIC frequency ranging from 15 to 50% in multiple independent tumors in all three models [17]. Moreover, tumors arising from transplanted microscopically identified single tumor cells recapitulated the histology and cellular composition of the mammary tumors from which they were originally isolated. Culture of the primary mouse mammary tumor cells in chemically defined, serum-free media as non-adherent spheres, termed tumorspheres [18], preserved the BTIC frequency of the primary tumors from which they were established [17]. The serum-free media was first developed to propagate mouse neuronal stem cells, termed neurospheres, and it is now commonly used to culture stem cells from diverse tissues and organs [19]. By contrast, propagating the primary tumor cells in serum-containing media as adherent populations resulted in a decrease in their BTIC frequency by 4–5 orders of magnitude [17].

### 2.2. Phenotypic Screen in Mouse BTIC-Enriched Tumor Cells Identifies Neurotransmitter Antagonists

The availability of tumors bearing high BTIC frequencies enabled a phenotypic screen to identify small molecules that targeted these cells. Tumorsphere-derived cells from the mammary tumors of an MMTV-Neu transgenic mouse strain [20] were used as a source of BTIC-enriched populations, and alamarBlue was used to assess the capacity of approximately 35,000 small molecules to affect their viability [21]. All the hits were counter screened using mouse mammospheres (primary mouse mammary epithelial cells) derived from the same mouse strain (FVB/N) as the transgenic mice. Compounds that were generally toxic in both tumorspheres and mammospheres were excluded from further analyses.

Unexpectedly, inhibitors of neurotransmitter activity were identified among the 3500 bioactive compounds of known mechanisms of action including approved drugs [22]. The latter included selective serotonin and dopamine reuptake inhibitors that target their transporters, and serotonin, dopamine, cannabinoid, opioid, histamine, and adrenergic receptor antagonists. All the receptors identified in the screen are members of the G-protein receptors (GPCRs) superfamily. Notably, the neurotransmitter antagonists were the third largest class of compounds after antibiotics and inhibitors of cell signaling, which collectively comprised 50% of all the bioactive hits.

The neurotransmitter antagonists included nine that affect the activity of serotonergic system proteins. The latter included three selective serotonin reuptake inhibitors (SSRI; Zoloft (sertraline), Prozac (fluoxetine) and Paxil (paroxetine)), which target the serotonin reuptake transporter (SERT), and other compounds that inhibit the activity of serotonin, (5-hydroxytryptamine; 5-HT), receptors (5-HTRs) with a half-maximal inhibitory concentration (IC_50_) in the low micromolar range. Given the high target selectivity of the antagonists and their clinically relevant IC_50_ in the in vitro assays, it seemed likely that their activity was not due to an off-target effect.

### 2.3. 5-HT and Proteins Required for Its Synthesis, Transport, and Activity via 5-HTRs Are Expressed in Mouse Mammary Tumor Cells

The enzymes required for 5-HT biosynthesis, transport, signaling, and catabolism are not restricted to the central and peripheral nervous systems. The latter are found in the gastro-intestinal tract, the cardiovascular system, and the breast. Indeed, enterochromaffin cells in the small intestine account for the synthesis of 90% of 5-HT in humans, which is stored in platelets.

Prolactin, which stimulates the expression of proteins found in milk during pregnancy and lactation, activates the expression of (tryptophan hydroxylase 1 (TPH1), required for 5-HT biosynthesis in mouse mammary secretory epithelial cells [23]). TPH1 possesses the same enzymatic activity as TPH2, a structurally related subfamily member, which is restricted to neuronal tissues. 5-HT activity in the mammary gland requires 5-HTRs because methysergide, a non-selective 5-HTR antagonist, blocks the effect of 5-HT. The expression of 5-HT pathway components, including, TPH1, monoamine oxidases (MAO-A and -B) required for 5-HT catabolism, SERT, and various 5-HTRs are expressed in mouse and bovine mammary glands [24,25]. The model to emerge from these findings is that prolactin induces the expression of TPH1, resulting in the synthesis of 5-HT, which acts via 5-HTRs, to induce the death of secretory mammary epithelial cells by apoptosis during lactation and involution in the mammary glands of both mice and cows. Interestingly, two different 5-HTRs have been identified that play distinct roles during lactation (5-HTR2B) and involution (5-HTR7) in mice [26].

### 2.4. 5-HT Is Synthesized by Mouse Mammary Tumor Cells, Which Express TPH1 and SERT

The finding that 5-HT plays an important role in postnatal mouse mammary gland development coupled with the fact that the machinery for 5-HT synthesis and function occurs in mouse mammary glands encouraged us to further explore the role of 5-HT in breast cancer and to determine whether SERT and 5-HTR selective antagonists targeted BTIC. There are numerous well-characterized and widely used drugs affecting 5-HT activity that are prescribed for mood disorders. Hence, if 5-HT signaling plays a critical role in BTIC activity, then one or more of these drugs might be repurposed to treat breast cancer in combination with standard therapies.

The latter was initially addressed by determining whether SERT is expressed in mouse mammary tumors from the MMTV-Neu transgenic mice. Immunofluorescence (IF) analyses of the sections of independent mouse mammary tumors revealed that SERT was indeed expressed in most of the tumor cells [22]. This result suggested that other proteins required for 5-HT synthesis and/or activity might be similarly expressed in mouse mammary tumors. Hence, mammary tumor sections from multiple tumors were stained with antibodies specific for TPH1 and 5-HT using the IF assay format. Nearly all the tumor cells expressed TPH1 and produced 5-HT. Hence, mouse mammary tumors possess the capacity to synthesize and transport 5-HT, implying that it plays a functional role in the tumor cells.

### 2.5. Serotonergic Antagonists Target BTIC from a Mouse Model of HER2-Overexpressing Breast Cancer

To learn whether the serotonergic antagonists affected the frequency of BTIC, quantitative sphere-forming assays, a functional in vitro assay for both mammary epithelial stem cells (MESC) and BTIC, were performed [11,27,28]. Independent studies have shown that MESC [29] and BTIC co-fractionate with sphere-forming cells after fluorescent-activated cell sorting [30,31], and that agents that alter MESC/BTIC frequency similarly affect the frequency of sphere-forming cells, suggesting that MESC/BTIC possess sphere-forming capacity [17,32,33]. Studies have also demonstrated that when seeded at clonal densities, tumorspheres arise directly proportional to the number of cells plated into the media and that the frequency of sphere-forming cells can be accurately quantified [22,33]. Indeed, single primary mouse mammary tumor cells form tumorspheres at the same frequency as those seeded at higher densities.

Three selective SERT antagonists (sertraline, fluoxetine, and paroxetine) identified in the screen reduced the frequency of sphere-forming cells in a dose-dependent fashion in multiple independent tumorsphere cultures derived from MMTV-Neu mammary tumors of independent mice, suggesting that they targeted BTIC [22]. Selective antagonists of TPH-1 and those of 5-HTR1B, 5-HTR2C, 5-HTR5A, and 5-HTR6 also targeted BTIC using sphere-forming assays. It is of relevance that whereas the chemically defined media used to culture tumorspheres does not contain 5-HT, it does include tryptophan, which is an essential amino acid and substrate of TPH1. Hence, any 5-HT detected by IF must have been synthesized by the tumor cells.

To confirm that the serotonergic antagonists indeed targeted BTIC, ex vivo assays, the gold standard assay for tumor-initiating cells (TIC) [5] were performed. In short, tumorsphere-derived cells were incubated with multiple doses of sertraline in vitro. Thereafter, the cells were orthotopically transplanted into immune-competent FVB/N mice, which is the same strain as the transgenic mice. Exposure of the cells to sertraline delayed the onset and growth rate of the tumor allografts and reduced their volume at endpoint in a dose-dependent manner [22] in keeping with the SSRIs targeting BTIC [17]. It is noteworthy that we [17] and others [34] have shown that time to tumor allograft or xenograft onset, growth rate, and volume at endpoint are directly proportional to the frequency of BTIC in the transplanted tumor cell population. 

### 2.6. Serotonergic Antagonist Function by an Irreversible Mechanism to Reduce BTIC Frequency

To learn whether the selective antagonists functioned by a reversible or irreversible process to reduce BTIC frequency, secondary sphere-forming assays were performed [22]. In short, tumorsphere-derived cells from multiple mouse mammary tumors were incubated independently with the vehicle or with each of three SSRIs at multiple doses. Thereafter, the cells were dissociated from the tumorspheres that formed and reseeded into chemically defined SSRI-free media required for sphere formation. Then, the frequency of the tumorspheres that arose in each of the cultures was determined.

The frequency of sphere-forming cells in the cultures incubated with the vehicle was the same as that in the primary and secondary sphere-forming assays. By contrast, the frequency of the tumorspheres that appeared in the cultures incubated with each of the SSRIs was reduced in a dose-dependent fashion in the secondary assay. These observations demonstrate that the SSRI induced an irreversible process in the BTIC, perhaps differentiation or cell death, resulting in their loss of tumorigenicity.

### 2.7. Serotonergic Antagonists Synergize with Chemotherapy to Shrink Mouse Mammary Tumors

Clinical trials to assess the activity of an SSRI as an anticancer agent would likely be carried out by treating terminal breast cancer patients with both an SSRI and a cytotoxic therapy. Moreover, achieving long-lasting breast cancer remissions will require targeting both the BTIC and the non-tumorigenic tumor cells, which are a potential source of BTIC [11]. Hence, mice bearing established mammary tumor allografts were treated with the vehicle, sertraline, docetaxel (a commonly used cytotoxic drug to treat breast cancer), and the combination of both drugs. Tumor growth rate and final volume at endpoint were monitored over the course of several weeks. Both sertraline and docetaxel independently reduced tumor growth rate and tumor volume at endpoint [22]. However, the combination of both compounds reduced both parameters to a much greater extent than each drug did individually, suggesting that they functioned synergistically.

### 2.8. Sertraline in Combination with Docetaxel Reduces Tumor Cell Proliferation and Induces Programmed Cell Death in Mammary Tumor Allografts

To determine the mechanism whereby the drugs functioned to slow tumor growth rate and to shrink mammary tumors, sections of the tumors were stained with antibodies to markers of proliferation (Ki67) or to those of apoptosis (terminal deoxynucleotidyl transferase dUTP nick end labeling (TUNEL)). Sertraline by itself reduced the frequency of Ki67-positive cells by about 50%, whereas docetaxel had little effect [22]. The combination of both drugs substantially reduced both the frequency of proliferating cells and increased that of apoptotic cells. These findings are consistent with the hypothesis that sertraline targets BTIC whereas docetaxel targets the non-tumorigenic tumor cell population and that the drugs act synergistically to shrink mammary tumors.

### 2.9. Summary

Collectively, the experimental findings described above strongly suggested a required role for 5-HT and effectors of its synthesis, transport, and 5-HTR-mediated signaling in mouse mammary tumor cells from the transgenic strain modeling HER2-overexpressing breast tumors. Whether these observations were of relevance to human breast tumors or to specific breast cancer subtypes remained unaddressed.

## 3. 5-HT and Human Cancers

The first evidence that GPCRs play a role in human cancers was revealed by the identification of the Mas oncogene from a human epidermoid carcinoma using DNA-mediated transfer of genomic DNA into mouse 3T3 cells and their subsequent transplantation into nude mice to generate xenografts [35]. Molecular cloning and sequencing of the human DNA from the xenografts revealed that Mas is a member of the GPCR superfamily.

Subsequently, three different 5-HTRs (5-HTR1A, 5-HTR2A, and 5-HTR2C) were found to be capable of transforming mouse 3T3 cells in vitro using focus forming assays [36,37,38]. 5-HTR-mediated focus formation required 5-HT, and antagonists of the 5-HTRs blocked this process. Agonist-dependent oncogenic transformation of 3T3 or Rat-1 cells has also been demonstrated for several other GPCRs [39,40]. In these examples, overexpression of the wild-type GPCR elicits ligand-dependent murine fibroblast transformation.

Mutations in GPCR that render them ligand-independent have also been identified by DNA sequencing of human tumor samples [41,42]. In fact, approximately 20% of human tumors possess mutated GPCRs or Gα subunits, which mediate signal transduction via cyclic AMP (cAMP). Moreover, increased copy number alterations and increased expression of genes encoding a subset of 5-HTRs, Gα subunits [41,42], and SERT [43] have been identified in breast and other tumors. The fact that 5-HTRs and their downstream effectors have been implicated in different cancers raises the prospect that their antagonists may be candidates for anticancer drug development [44,45].

Interestingly, the overexpression of 5-HTRs in human melanoma cell lines enhances their resistance to kinase inhibitors targeting BRAF or MEK [46,47,48]. The latter findings support a link between 5-HTRs, kinases, and TIC, which are resistant to various anticancer therapies such as those that target kinases. Moreover, query of the cMAP database with a recently published stemness gene signature identified 17 drugs that target three different 5-HTRs, suggesting that these receptors may play a role in stem cell self-renewal [49].

It is also noteworthy that plasma-free 5-HT in serum is a biomarker of early breast cancer recurrence [50] and that active 5-HT synthesis is a metabolic feature of tumors of breast cancer patients with a poor prognosis [51]. Taken together, these observations suggest that 5-HTRs or their downstream effectors can function as oncogenes to stimulate signaling pathways active in breast tumors and that their selective antagonists may enhance the sensitivity of 5-HT producing breast tumors to kinase inhibitors.

### 3.1. Human Breast Tumors, Patient-Derived Xenografts and Breast Tumor Cell Lines Synthesize 5-HT and Express Proteins Required for Its Biosynthesis and Activity

Studies have revealed that 5-HTR2A is expressed in the MCF-7 breast tumor cell line [52], whereas TPH1 and SERT are expressed in the MCF-7, MB-MDA-231, and T47D breast tumor cell lines [53]. TPH1 and 5-HTR1A, -1B, -2B, -4, 5A, and -7 are also expressed in human breast tumors [53,54,55]. Table 1 summarizes the relevant literature pertaining to 5-HT-related gene expression. 5-HT modestly stimulates the proliferation of MCF-7 cells [52] and induces an epithelial to mesenchymal transition in MB-MDA-231 cells [56].

Antibodies specific to SERT, TPH1, or 5-HT were used to learn whether they are present in human breast tumor cell lines, breast tumors, and their xenografts. Western immunoblotting revealed that SERT was expressed in each of eight breast tumor cell lines, modeling all the subtypes of breast cancer [43]. TPH1 and 5-HT were also present in the cells comprising tumorspheres derived from the HCC1954 breast tumor cell line and those of its xenografts using IF. Immunohistochemistry (IHC) detected SERT in six sections of primary human breast tumors and their corresponding xenografts independent of their subtype. Interestingly, a minor subpopulation of the tumor cells in each xenograft expressed SERT at much higher levels compared to surrounding tumor cells. Whether the SERT overexpressing tumor cells represent BTIC is not known. Hence, human breast tumor cells propagated in vitro as tumorspheres, and primary breast tumors and their xenografts possess the capacity to synthesize and transport 5-HT independent of their subtype.

### 3.2. Selective Antagonists of Multiple 5-HT System Pathway Components Reduce BTIC Frequency in Human Breast Tumor Cell Lines and Synergize with Chemotherapy to Shrink Breast Tumor Xenografts

**Figure 2:**
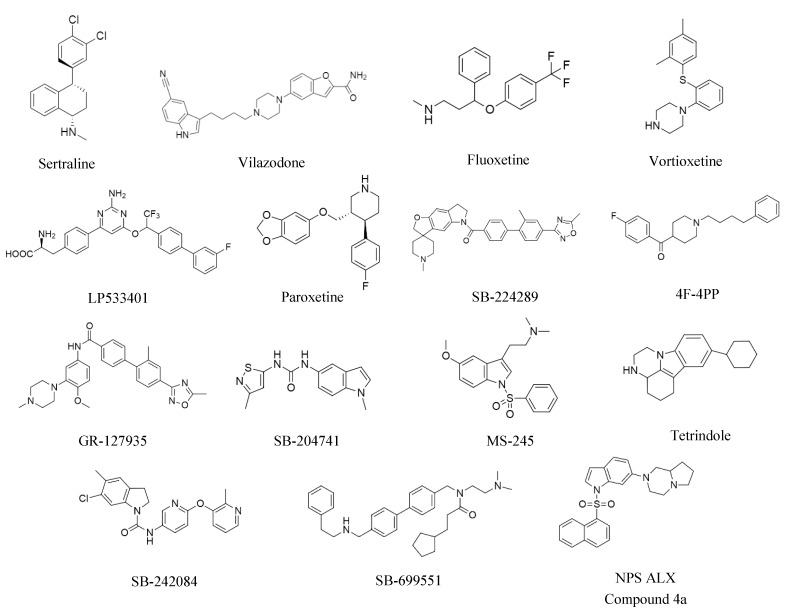
Chemical structures of serotonergic antagonists investigated.

To learn which 5-HT pathway proteins are required for human BTIC survival, the activity of those selective 5-HT antagonists that were commercially available was evaluated in up to 10 human breast tumor cell lines modeling all breast cancer subtypes using alamarBlue cell viability and quantitative sphere-forming assays. Tumorsphere-derived cells from all the cell lines were equally sensitive to selective antagonists of various serotonergic pathway proteins as evaluated using both assays [43]. Notably, selective antagonists of TPH1, SERT, and a subset of 5-HTRs (5-HT1B, 5-HT1D, 5-HT2A, 5-HT2B, 5-HT2C, 5-HT5A, and 5-HT6) all reduced tumor cell viability and tumorsphere-forming cell frequency in all the breast tumor cell lines. The chemical structures of the various serotonergic antagonists are shown in Figure 2. 5-HT3 selective antagonist did not affect the viability or frequency of sphere-forming cells. Notably, the 5-HT3 family members are not GPCRs but ligand-gated channels. Other 5-HTRs that might be required for tumor cell viability and sphere formation were not assayed because their selective antagonists were not commercially available. However, a novel 5-HT7 selective antagonist was recently reported to affect the proliferation and migration of several breast tumor cell lines modeling TNBC [55].

**Figure 3:**
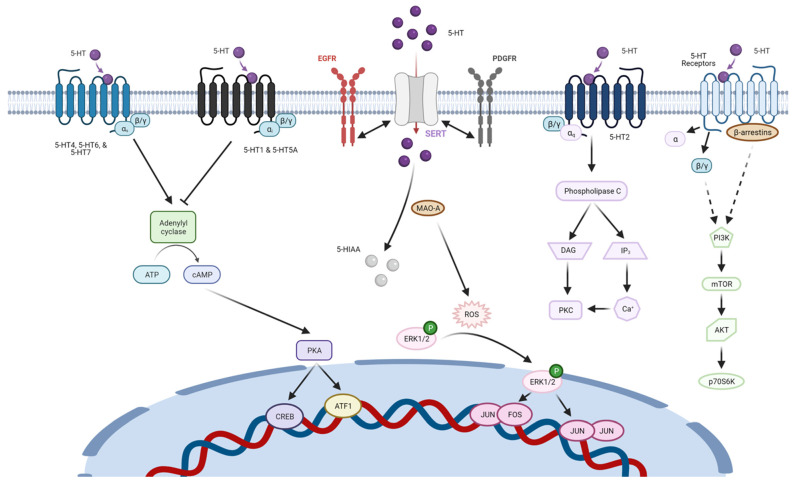
Overview of serotonergic signaling. Schematic displaying canonical and noncanonical signaling pathways coupled to serotonergic signaling proteins.

Whether the numerous 5-HTRs identified above perform redundant or unique functions required for BTIC survival or sphere formation is not known. The fact that two different 5-HTRs fulfill distinct roles during postnatal mouse mammary gland development suggests that 5-HTRs may similarly fulfill different functional roles in human breast tumors [26,60]. In this regard, it is notable that the 5-HTRs known to be required for BTIC activity signal via Gα subunits that either increase or decrease cAMP levels, suggesting that cAMP-mediated signaling may not play a role in regulating BTIC activity (Figure 3) [43]. Instead, the effectors of 5-HTR signaling mediated by Gβγ subunits and/or those affected by β-arrestins, which serve as a scaffold for a number of kinases, may play a required role in BTIC [61,62]. It is notable that SERT, whose selective antagonists target BTIC, share the capacity to signal via receptor tyrosine kinase (RTK)-mediated pathways that are also downstream of 5-HTRs (Figure 3) [63].

Ex vivo assays carried out by incubating human breast tumorsphere-derived cells of the HCC1954 human breast tumor cell line with the vehicle or sertraline in vitro followed by the transplantation of equal numbers of viable cells into immune-compromised mice in vivo resulted in a delay in the onset of breast tumor xenografts and a reduction in their volume at end point, which is consistent with sertraline reducing BTIC frequency [43]. Moreover, an SSRI (vilazodone (Viibryd)) synergized with chemotherapy to substantially reduce the growth rate and final volume at endpoint of HCC1954 breast tumor xenografts.

Analyses of the effect of the drugs independently and in combination revealed that individually, they did not have a major effect on tumor cell proliferation or apoptosis, but their combination reduced tumor cell proliferation and increased apoptosis. These observations coupled with those from the experiments performed in mice bearing mouse mammary tumor allografts suggest that the combination of a serotonergic antagonist targeting BTIC and a cytotoxic therapy targeting the non-tumorigenic tumor cells may provide more longer-lasting breast cancer remissions than are achieved currently.

### 3.3. Functional Evidence for 5-HTRs in Human Breast Cancer and Other Cancers

In keeping with the finding that serotonergic antagonists affect the activity of BTIC in mouse and human models of breast cancer, several studies have demonstrated that genetic inhibition of TPH1 or 5-HTRs have a wide range of consequences on tumor cell phenotypes [55,64]. A doxycycline-induced CRISPR-cas9-mediated knockout of 5-HTR5A in human breast tumor cell lines impairs their capacity to form tumorspheres in vitro and their engraftment into nude mice in vivo [64]. Other studies have shown that genetic knockdown (KD) of TPH1 reverses 5-HT induced proliferation and invasion by MDA-MB-231 triple negative breast cancer cells in vitro [55]. KD of 5-HTR7 similarly reduces the invasive properties of the tumor cells. Interestingly, in the latter study, 5-HT synthesized by TPH1 promoted vascular endothelial growth factor (VEGF) signaling, which is crucial to early tumor initiation and growth.

A functional role for 5-HTRs in human cancers and cancer stem cells is not exclusive to breast cancer, and the genetic depletion of 5-HTRs has deleterious consequences in several tumors of other solid organs and hematological malignancies [65,66,67,68]. For example, KD of 5-HTR1B and 5-HTR1D inhibits the proliferation and clonogenic capacity of pancreatic tumor cells by inhibiting the activity of transcription factors involved in initiating an EMT [68]. Similarly, KD of 5-HTR1B dampens the proliferation, migration, and invasive properties of non-small cell lung carcinoma [67]. In acute myeloid leukemia (AML), 5-HTR1B has a functional role in maintaining therapy-resistant leukemic stem cells [66]. Hence, 5-HTR1B has been proposed as a therapeutic target for AML, myelodysplastic syndromes, and chronic myelomonocytic leukemia [65]. Of note, one of the tool compounds used to inhibit 5-HTR1B in the latter studies, SB-224289, also demonstrated efficacy against BTIC (Table 2) [22,43]. Taken together, these studies provide complementary genetic evidence for the role of 5-HTRs and TPH1 in breast cancer and other human cancers.

## 4. Monoamine Oxidase A (MAO-A)

To learn whether 5-HT stimulates breast tumor cell processes such as cell proliferation, migration, and sphere formation, exogenous 5-HT or 5-carboxamidotryptamine (5-CT) was added to the chemically defined media used to culture breast tumor cell lines as tumorspheres. 5-CT acts as a non-selective high-affinity agonist of most 5-HTRs [69]. The chemically defined serum-free media does not contain 5-HT. Whereas 5-HT and 5-CT did increase tumor cell survival and the frequency of sphere-forming cells, the magnitude of these effects was less than 2-fold (unpublished).

Hence, we wondered whether blocking 5-HT turnover might provide an alternative and more robust means to achieve high intracellular 5-HT levels leading to a greater effect on breast tumor cell processes. To this end, selective antagonists of the monoamine oxidases, MAO-A and MAO-B, which catabolize 5-HT, dopamine, and norepinephrine were used with the expectation that inhibition of their abundance would increase intracellular 5-HT levels. MAO-A or -B are outer mitochondrial membrane flavoenzymes, which use oxygen as a cofactor to deaminate monoamine neurotransmitters producing free reactive oxygen radicals as a by-product [70].

In advance of performing mechanistic studies with the antagonists, we determined whether MAO-A transcripts and protein were expressed in a panel of 10 human breast tumor cell lines comprising all clinical subtypes. MAO-A was not expressed in the majority of breast tumor cell lines when they were propagated in serum-containing media: however, its abundance at both the RNA and protein levels was dramatically increased when the tumor cells were cultured in chemically defined media as tumorspheres [57]. Hence, in vitro culture conditions conducive for maintaining a high BTIC frequency stimulates MAO-A expression.

Consequently, MAO-A and MAO-B selective antagonists were evaluated using cell viability and sphere-forming assays. By contrast to our expectations, three selective MAO-A antagonists tested reduced cell viability and the frequency of sphere-forming cells derived from the two breast cancer cell lines (MCF-7 and HCC1954) that were tested [57]. MAO-B selective antagonists had no effect in the latter assays. These findings suggested that MAO-A activity is in fact required to maintain BTIC survival and their capacity to form breast tumorspheres.

In silico mining of gene expression profiles of breast tumor cell lines that overexpressed individual components of RTK signaling pathways revealed that epidermal growth factor, a component of the chemically defined media required for sphere formation, increased the abundance of MAOA transcripts [57]. Interestingly, MAOA transcript expression was also positively associated with resistance of breast tumor cell lines to both cytotoxic and targeted anticancer therapies. Moreover, like the expression of BTIC markers such as aldehyde dehydrogenase [71] and CD44 [72], increased MAOA transcripts expression is associated with reduced relapse-free survival in high-grade breast tumors of the TNBC clinical subtype [57]. These findings further strengthened the link between BTIC and MAO-A activity.

The role of MAO-A in cancer is controversial. Whereas some studies have suggested that loss of MAO-A expression accompanies oncogenic transformation in many solid tumors [73], others have reported that increased MAO-A activity facilitates tumorigenesis, EMT, and resistance to cytotoxic anticancer therapies, which are processes that support a required role for MAO-A in BTIC [57,74,75].

Several studies have suggested that SERT and MAO-A can function in concert to potentiate mitogenic signaling pathways downstream of 5-HTRs. For example, 5-HT-mediated stimulation of 5-HT1B in bovine pulmonary smooth muscle cells results in increased proliferation and migration via the phosphorylation of extracellular signal-regulated kinases (ERKs) [76]. MAO-A mediated deamination of 5-HT transported intracellularly by SERT generates reactive oxygen species (ROS) that facilitate nuclear translocation of phospho-ERK, resulting in changes in the activity of transcription factors (Figure 3). Similar cooperative interactions between 5-HTR2B, MAO-A, and SERT have been observed in cardiac myocytes [77]. Hence, the mitogenic effects mediated by 5-HT on 5-HTRs in peripheral tissues such as the breast requires ROS produced by MAO-A-mediated deamination of 5-HT.

## 5. Conclusions and Future Directions

In summary, 5-HT is produced in breast tumors of mouse and human origin as well as in cell lines derived from these sources, and inhibition of its biosynthesis, transport, signaling activity via 5-HTRs, and catabolism all resulted in a reduced frequency of BTIC, as measured by multiple orthologous assays. Furthermore, those selective serotonergic antagonists tested synergized with docetaxel to shrink both mouse mammary tumor allografts and human breast tumor xenografts by inhibiting tumor cell proliferation and inducing apoptosis (findings summarized in Table 2). A common feature of the various serotonergic system antagonists selectively targeting many different serotonergic pathway proteins is their capacity to attenuate the activity of downstream kinases-mediated pathways that are dysregulated in tumors of diverse origin, including those of the breast. Hence, agents that affect 5-HT mediated processes via a number of different serotonergic pathway components may be excellent candidates for anticancer drug development.

The research findings summarized herein illustrate a compelling rationale for further investigation of the serotonergic signaling system in human breast cancer with the objective to develop more effective therapeutic agents. A deepened molecular understanding of the functional role of 5-HT in tumor cells by comparison to normal cells will be crucial to achieve the latter goal. Moreover, a holistic view of the signaling events that occur downstream from serotonergic proteins after their inhibition or activation might expand the repertoire of existing or candidate drugs to treat breast cancer. More extensive loss-of-function and gain-of-function studies will help shed light on therapeutic vulnerabilities that might aid anticancer drug development.

The notion of 5-HT as a biomarker for cancer recurrence is also gaining momentum in recent years. A recent study implicated plasma-free 5-HT as a biomarker of metastatic colorectal carcinoma [78], which is like the findings in breast cancer patients recounted above. Hence, assessment of circulating 5-HT concentrations in the plasma of patients diagnosed with breast or colon cancer might serve as a valuable clinical tool in the future to stratify patient risk and to identify patients who might benefit from the use of drugs that target serotonergic system proteins.

## Figures and Tables

**Table 1:** Summary of serotonergic proteins expressed in breast cancer.

Protein		Sample Type	Detection Method	Reference
SERT		MMTV-Her2/Neu tumorspheres and tumors	IF	[22,43,56]
		Patient-derived breast tumors xenografts	IHC	
		Human breast tumor cell lines, tumorspheres and xenografts	WB, IF, RT-PCR	
TPH1		MMTV-Her2/Neu tumorspheres and tumors	IF	[22,43,55,56]
		Human breast tumor cell lines, tumorspheres and xenografts	IF	
5-HT		MMTV-Her2/Neu tumorspheres and tumors	IF	[22,43]
		Human breast tumor cell line tumorspheres and xenografts	IF	
MAO-A		Human breast tumor cell lines and tumorspheres	NanoString, WB	[57,58,59]
		Chemically induced rat mammary tumors	Enzymatic	
5-HTR	1A 1B 2A 2B 4 5A 7	Human breast tumor cell lines	DNA microarray, RT-PCR, IF, WB	[52,53,54,55,56]

Immunofluoresence (IF); Immunohistochemistry (IHC); Western blotting (WB); Reverse Transcription Polymerase Chain Reaction (RT-PCR).

**Table 2:** Summary of pharmacological findings.

Molecule	FDA-Approved	Target	Sample Type	Species	Observed Effects
LP533401	No	TPH1	MMTV-Her2/Neu	Mouse	Inhibits tumorsphere formation [22,43]
			Breast tumor cell lines	Human	
Sertraline	Yes	SERT	MMTV-Her2/Neu	Mouse	Inhibits tumorsphere formation [22]
					Targets tumor-initiating cells [22]
					Induces tumor regression in combination with docetaxel [22]
			Breast tumor cell lines	Human	Inhibits tumorsphere formation [43]
					Targets tumor initiating cells [43]
Paroxetine	Yes	SERT	MMTV-Her2/Neu	Mouse	Inhibits tumorsphere formation [22,43]
			Breast tumor cell lines	Human	
Fluoxetine	Yes	SERT	MMTV-Her2/Neu	Mouse	Inhibits tumorsphere formation [22,43]
			Breast tumor cell lines	Human	
Vortioxetine	Yes	SERT	Breast tumor cell lines	Human	Inhibits tumorsphere formation [43]
Vilazodone	Yes	SERT, 5-HTR1A	Breast tumor cell lines	Human	Inhibits tumorsphere formation [43]
					Induces tumor regression in combination with docetaxel [43]
SB-224289	No	5-HTR1B	MMTV-Her2/Neu	Mouse	Inhibits tumorsphere formation [22,43]
			Breast tumor cell lines	Human	
GR-127935	No	5-HTR1D	Breast tumor cell lines	Human	Inhibits tumorsphere formation [43]
4F-4PP	No	5-HTR2A	Breast tumor cell lines	Human	Inhibits tumorsphere formation [43]
SB-204741	No	5-HTR2B	Breast tumor cell lines	Human	Inhibits tumorsphere formation [43]
SB-242084	No	5-HTR2C	Breast tumor cell lines	Human	Inhibits tumorsphere formation [43]
SB-699551	No	5-HTR5A	MMTV-Her2/Neu	Mouse	Inhibits tumorsphere formation [22,61]
			Breast tumor cell lines Patient-dervied breast tumor xenografts	Human	Inhibits tumorsphere formation [43]
					Targets tumor-initiating cells [61]
					Induces tumor regression in combination with docetaxel [61]
MS-245	No	5-HTR6	MMTV-Her2/Neu	Mouse	Inhibits tumorsphere formation [22]
NPS ALX Compound 4a	No	5-HTR6	Breast tumor cell lines	Human	Inhibits tumorsphere formation [43]
Tetrindole	No	MAOA	Breast tumor cell lines	Human	Inhibits tumorsphere formation [57]

## Data Availability

The only unpublished data recounted in the manuscript is available on request from the corresponding author.

## Abbreviations

Abbreviation	Definition
5-HT	5-Hydroxytryptamine
WB	Western blot
5-HTRs	5-HT receptors
TNBC	Triple-negative breast cancer
ER	Estrogen receptor
PR	Progesterone receptor
BTICs	Breast tumor-initiating cell
EMT	Epithelial to mesenchymal transition
GPCRs	G-protein coupled receptors
SERT	Serotonin reuptake transporter
5-HT	5-hydroxytryptamine
SSRI	Selective serotonin reuptake inhibitors
TPH1	Tryptophan hydroxylase 1
MAO	Monoamine oxidase
MESC	Mammary epithelial stem cells
TIC	Tumor-initiating cells
TUNEL	Terminal deoxynucleotidyl transferase dUTP nick end labeling
IHC	Immunohistochemistry
IF	Immunofluorescence
RTK	Receptor tyrosine kinase
5-CT	5-carboxamidotryptamine
ERKs	Extracellular regulated kinases
cAMP	Cyclic adenosine monophosphate
